# Improving Outcomes from Breast Cancer in a Low-Income Country: Lessons from Bangladesh

**DOI:** 10.1155/2012/423562

**Published:** 2011-12-05

**Authors:** H. L. Story, R. R. Love, R. Salim, A. J. Roberto, J. L. Krieger, O. M. Ginsburg

**Affiliations:** ^1^International Breast Cancer Research Foundation, 660 John Nolen Drive, Madison, WI 53713, USA; ^2^School of Human Evolution and Social Change, Arizona State University, P.O. Box 872402, Tempe, AZ 85287-2402, USA; ^3^Amader Gram, 11/8 Iqbal Road, Ground Floor, Dhaka 1207, Bangladesh; ^4^Hugh Downs School of Human Communication, Arizona State University, P.O. Box 871205, Tempe, AZ 85287, USA; ^5^The Ohio State University, 3058 Derby Hall, 154 North Oval Mall, Columbus, OH 43210-1339, USA; ^6^Department of Medicine, Women's College Research Institute, University of Toronto, 790 Bay Street, 7th Floor, Toronto, ON, Canada M5G 1N8

## Abstract

Women in low- and middle-income countries (LMICs) have yet to benefit from recent advances in breast cancer diagnosis and treatment now experienced in high-income countries. Their unique sociocultural and health system circumstances warrant a different approach to breast cancer management than that applied to women in high-income countries. Here, we present experience from the last five years working in rural Bangladesh. Case and consecutive series data, focus group and individual interviews, and clinical care experience provide the basis for this paper. These data illustrate a complex web of sociocultural, economic, and health system conditions which affect womens' choices to seek and accept care and successful treatment. We conclude that health system, human rights, and governance issues underlie high mortality from this relatively rare disease in Bangladesh.

## 1. Introduction

The US-based National Comprehensive Cancer Network guidelines for breast cancer management specifically state that even under the best of circumstances “there is not a single clinical situation in which the treatment of breast cancer has been optimized with respect to either maximizing cure or minimizing toxicity and disfigurement” [1]. In low- and middle-income countries with far fewer resources than the US, the circumstances are compounded by multiple factors associated with increased mortality for this disease [2]. Addressing and remedying these inequities requires an exploration into the unique circumstances surrounding the complex barriers women face in receiving information, accurate and timely diagnosis, and effective treatment critical to reducing breast cancer morbidity and mortality [3].

For the past five years, beginning with work to recruit women to a clinical trial of treatment for metastatic breast cancer, we have been increasing our efforts to understand what is happening to women with breast cancer in the Khulna Division of Bangladesh. Our experience calls into question the application of common high-income country models and strategies in such settings.

## 2. Bangladesh

Understanding barriers to improving outcomes from breast cancer begins with an appreciation of the broader sociocultural context in which women live. We present our experience in Bangladesh as the backdrop for this exploration.

**Figure 1:**
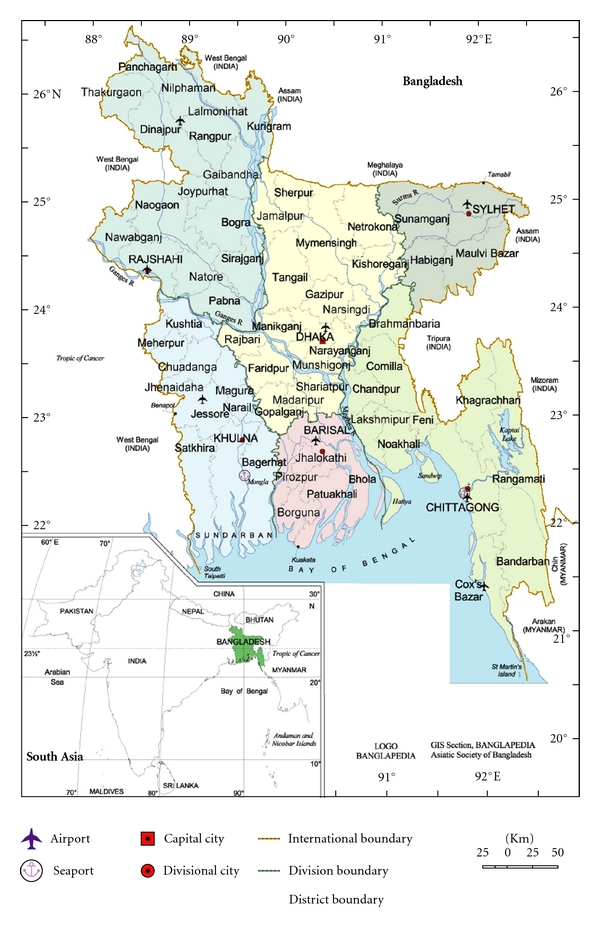
Map of Bangladesh and its six administrative divisions.

Bangladesh is located in Southern Asia, between India and Myanmar, and borders the Bay of Bengal to the south (Figure 1). It is the seventh most populous country in the world; a country of nearly 160 million people (approximately half the population of the US) in an area half the size of Italy or a mid-sized state in the US such as Iowa. Over 70% of the country is considered rural although population density is high throughout the country [4]. The country is administratively divided into six divisions, which are further divided into districts. The Khulna Division, which has been the focus of our work, has a population of 15.5 million [5].

Over 89% of Bangladeshis consider themselves Muslim, making Bangladesh the third largest Muslim-dominated country after Indonesia and Pakistan. Approximately 45% of the population is employed in the agricultural sector. Bangladesh is a low-income country, defined by the World Bank as countries with a Gross National Income (GNI) less than US $1,005 per capita [6]. About 40% of the population is underemployed; many participants in the labor force work only a few hours a week, at low wages [4]. Approximately 60% of women are illiterate [4], and 27% of the population is undernourished [7].

Primary health care is provided through government and nongovernment rural health clinics, with referrals to the District or Division level for secondary or tertiary level care. Tertiary health care, however, can rarely be received at the Division level or lower due to lack of trained health care providers, treatment facilities, and patient resources. In the case of radiation therapy, resources are significantly lacking. There are approximately eighteen functional radiation therapy units in Bangladesh when an estimated three-hundred such units are needed. Individuals seeking treatment such as that found in high-income countries must travel to the capital city, Dhaka, or leave the country if resources allow. For most rural-dwelling people in Bangladesh, this is simply not feasible, leaving them to rely on available local services. Many rural people first or exclusively seek care from a variety of alternative care for health problems, including ayurvedic, homeopathic, spiritual, and self-proclaimed healers [8, 9].

There is no system of national health insurance in Bangladesh. Government facilities charge nominal fees for admission, but the majority of medical expenses (diagnostics, surgery, medications, etc.) are paid for out-of-pocket. Furthermore, studies show that patients are often asked to pay more than the standard fees for services in order to receive priority treatment (such as a bed, or to see the doctor sooner), cleaning, access to scarce medications, or basic nursing [9–11].

Public hospitals are frequently overcrowded, unsanitary, and lacking in essential resources including basic equipment and essential drugs. Patients can be found sleeping on the floor or sharing beds, and report poor treatment by hospital staff and physicians [9–11]. Private hospitals with better resources are growing in number but are financially out of reach for most Bangladeshis.

The doctor-patient ratio in Bangladesh is 1 : 3,300 people [12] with 52% of doctors concentrated in urban areas (including private hospitals). One report estimated a tenfold difference, with 1 doctor for 1,500 people in urban versus 1 : 15,000 in rural areas [13]. This leaves rural doctors to manage extremely high caseloads. As in high-income countries, physicians are often unwilling to be posted in rural areas due to the lack of additional opportunities for private practice and preference for the conveniences of an urbanized area. Female doctors are even scarcer. Of all medical graduates since 1971, only 23% are female (Bangladesh Department of Health and Family Welfare, personal communication).

## 3. Breast Cancer Incidence and Mortality in Bangladesh

It is estimated that each year, 76,000 women die of breast cancer in South Asia (India, Bangladesh, Nepal, Myanmar, Pakistan, and Tibet) [14]. In Bangladesh, there is no national cancer registry. However, age-standardized incidence rates from Karachi, Pakistan (53.8/100,000) [15], and Kolkata, India (25.1/100,000) [16] (both with whom Bangladesh shares many cultural and historical similarities), suggest an annual incidence rate of 35–40/100,000. Therefore, in Bangladesh, we estimate an annual new breast cancer case burden of 30,000 women. The prevalence of breast cancer is expected to grow in South Asia due to a combination of increased life expectancy, population growth [17], and adoption of “Western” lifestyles (higher fat diets, reduced activity, reduced parity, delayed child bearing, and decreased breast feeding) [18]. It is projected that global breast cancer cases will grow from 1.4 million in 2008 to over 2.1 million cases in 2030 [19]. While high-income countries celebrate significant progress toward curing women with breast cancer, low-income countries like Bangladesh are only beginning to recognize the extent and severity of the disease.

## 4. The Case for Research

Research to improve health systems is particularly needed in the countries of South Asia, where the “triple burden” of communicable disease, chronic disease, and excess injury and violence drain already limited health resources [20]. The World Health Organization has repeatedly focused on primary care and health systems in reducing health disparities and improving global health in its recent reports [21]. Harford [22] has stressed in particular the importance of studying and addressing barriers which delay women from seeking care in low- and middle-income countries, and barriers within the health system which prevent or delay diagnosis of the disease, both of which contribute to advanced stage disease. An understanding of these barriers allows for the development of interventions which are culturally sensitive and more effective [3], in this case for the reduction of breast cancer mortality. Given the growing case burdens in low- and middle-income countries, the large number of women affected by the disease and the excessive mortality experienced by Bangladeshi women and South Asian women in general, studies which inform the development of better breast cancer management in the region are certainly warranted. As in many low-income countries, where cancer registries do not exist, hospital-based data are also often incomplete, and individual patient records may be sequestered within private clinicians' chambers. In light of these limitations, we present as a “case study” our group's efforts to understand the burden of breast cancer and suggest opportunities to improve the situation for women with breast cancer in Khulna Division, Bangladesh.

## 5. From the Ground Up: Exploring the Breast Cancer Situation in Bangladesh

The goal of The International Breast Cancer Research Foundation (IBCRF) is to end the suffering and death caused by breast cancer through “practical, cost-effective breakthrough research that takes advantage of the most promising opportunities and ideas everywhere in the world” [23]. IBCRF initiated participation in an international multicenter breast cancer clinical trial in Bangladesh in 2006 in collaboration with the major governmental hospital, Dhaka Medical College and Hospital, and later with other public and private hospitals including Khulna Medical College and Hospital in the city of Khulna, Bangladesh, and with the approval of research ethics boards in both the US and Bangladesh. Early in the process of implementing the clinical trial, we identified challenges to recruitment, including sobering anecdotes that for some women, a diagnosis of breast cancer was sufficient cause for divorce, rejection by the community, and in extreme cases a motivation for suicide; the consequences of diagnosis perhaps worse than the disease itself. Concordantly, the number of women presenting with breast problems at our collaborating medical facilities was lower than initially expected, and those who did come were wary that the proposed treatment was right for them. It became clear that a strictly hospital-based approach to our clinical trial in Bangladesh would be destined to fail. Furthermore, the potentially serious sociocultural implications to a breast cancer diagnosis necessitated that we should take a more extensive approach.

IBCRF and its partnering nongovernmental organization in Bangladesh, Amader Gram (“Our Village”), responded by venturing outside the hospital walls and leaving the capital city of Dhaka to explore the context in which women of rural Bangladesh experienced breast cancer. We focused primarily on the Khulna Division, where Amader Gram had previously established a trusted reputation as a health educator and pioneer in “information technology for rural development” (ICT4D), thus paving the way for us to engage with the community relatively quickly and economically. We created opportunities to interact with health professionals, government officials, community members, and women dealing with breast problems, which in turn allowed us to (1) efficiently address immediate social and health system concerns and (2) identify important research questions by which to mobilize resources and implement collaborative studies.

Our first efforts were in obtaining a better picture of the usual circumstances at presentation and diagnosis of breast cancer in our rural setting (Table 1). We reviewed 238 consecutive breast cancer case records at the tertiary care government hospital, Khulna Medical College Hospital. Cases were confirmed through either tissue diagnosis or clinically obvious breast cancer seen over a two-year period. Staging procedures were usually confined to a chest X-ray and an abdominal ultrasound examination. The largest number of cases has been categorized as Stage III+. The majority of these women had large tumors with associated regional adenopathy; tumors or lymph nodes were fixed to underlying structures. One quarter of these women had undergone lumpectomy surgery within the last six months with no histopathological examinations of removed tissues, and no additional treatments. Their presentations were often of kinds rarely seen in high-income countries today: mountainous infected and bleeding growths on their chest walls or in their axillae. While these data are not population based and the staging definitions and investigations used may differ from those employed in high-income and other countries, these data reinforce other information suggesting that in such low- and middle-income country circumstances, the overwhelming majority of women present to allopathic care givers with incurable disease. Indeed, genuinely palliative strategies in such circumstances are undefined.

 IBCRF and AG also sought to understand the estrogen hormone receptor status of the tumors of patients in the Khulna Division. Doctors reported that the tumors of most women in Bangladesh were estrogen hormone receptor negative. In this situation, we studied a small group of premenopausal patients with clinical circumstances strongly suggesting the presence of estrogen hormone receptor positive tumors: in this group, only 21% of tumors (3 of 14) were found to demonstrate receptors when evaluated by a surgical pathologist with rigorous procedures (Table 2). On the basis of work IBCRF had done in the Philippines, we implemented a set of tissue collection guidelines which called for prompt tissue fixation (within 30 minutes), duration of fixation over 8 hours, and use of buffered formalin fixative [24]. Subsequently, 72% (47 of 65) of tested tumors in patients expected to have hormone receptor positive tumors were found positive. These and other results suggest that Bangladeshi women have tumoral hormone receptor status similar to those of women in high-income countries. Thus, implementation of simple guidelines has led to treatment options for many women in Bangladesh who would have otherwise been assumed to have tumors insensitive to hormonal change and in fact never would have been offered the chance to have their tumors tested.

Next, we endeavored to learn more about breast cancer in rural Bangladesh by engaging the community in a variety of ways. Our efforts were based in four areas spread throughout the Khulna Division including Jessore city, Sreefaltola village in Bagerhat District, Rampal town, and Khulna city. We conducted a series of individual, semistructured interviews (*n* = 12), focus groups (2 total, *n* = 25), and community meetings (3 total, *n* = 29) with the aim of learning more about how women and their families deal with breast problems. Initially, female family members of staff and their friends participated in interviews. Later, convenience samples of women attending rural breast examination clinics supported by IBCRF were recruited to discuss their experiences. Women were asked questions to elicit their knowledge of breast cancer (what it is, signs and symptoms, and causes), where (or if) they would go for treatment, whether they knew someone with or currently were being treated for breast cancer, and perceptions of medical care for breast cancer. Community meetings consisted of key stakeholders including male and female government officers, journalists, local NGO employees, health professionals, and civil society opinion leaders. Meetings were jointly held by members of IBCRF and Amader Gram in both English and Bangla (the language of Bangladesh). A designated translator was present at all interviews and meetings to provide translation as needed. Community members were asked to discuss the status of breast cancer in their area, including their perceptions of the extent of disease, social stigma, treatment options, and possible solutions.

In addition, over 100 Khulna city doctors, including obstetricians and gynecologists, surgeons, radiotherapists, and hospital administrators were invited to participate in a discussion about breast cancer diagnosis and treatment in the Division. This created an opportunity for discussing Bangladesh's health system as it relates to breast cancer and resulted in numerous spin-off conversations which helped us understand the obstacles medical professionals face in obtaining good outcomes for their patients.

## 6. Patient and Community Group Discussion Findings

Focus groups and interviews with patients and community group meetings raised three main themes: knowledge of the disease, access to services, and the practice of seeking care.

### 6.1. Knowledge about Breast Cancer

Focus group and interview data from women in four rural Khulna Division sites suggest that some women are misinformed or have no knowledge about breast cancer. In fact, in some rural areas in Bangladesh, there is *no word for breast cancer;* using the word “breast” publicly is not permissible. A number of women interviewed said they did not know what to do about their breast problem until they heard that a foreign doctor was visiting their village to check for breast problems; only then, they decided to come themselves. Some women were brought by friends who had previously attended and recommended the service. Additionally, there are misperceptions and myths about how breast cancer is caused, which can have serious social ramifications. For example, some women believe that breast cancer is caused by an evil spirit, or as a punishment for bad deeds. A number of women expressed that they felt breast cancer “is a death sentence” and that “no good treatment exists”.

*“Younger women are not shy. They will come for care. Older generations are shy.”*

*-Focus group participant in Rampal.*

For the most part, however, the women who participated (particularly younger women) were aware of breast cancer and the fact that it is a serious disease. Even women who were misinformed or had a limited understanding about what caused their disease were eager to be examined when given the opportunity to see a doctor who was accessible to them.

*“It is evil. Once it visits your house it kills.”*

*“No one getting cancer gets saved.”*

*“It's a curse from God for wrong doings.”*

*-Focus group participants in Rampal.*

### 6.2. Access to Services

Women frequently reported that they could not access services for breast care. Reasons cited include the following: because they do not exist, they cannot get to services because their families will not allow them to leave the home, there is no money to pay for transport, the weather is inclement (Bangladesh has a monsoon season and is subject to typhoons which have devastated large areas in the past), or because road conditions are bad. In one instance, a woman discussed how she hired transport to go to the closest local doctor's office which was 45 minutes away, only to find that the doctor had not reported for duty that day. She noted that this was a common occurrence. Her valuable time and money was lost, and she was discouraged from seeking further care. During the holy month of Ramadan (a time of fasting and renewal of faith), women are less likely to leave the home in keeping with the ideal of “purdah,” where women are not typically seen in the public sphere. Clinic attendance drops each year during Ramadan, supporting this finding.

*“Nine out of ten women go to see a homeopath first. It costs much less.”*

*-Sreefaltola focus group participant.*

### 6.3. Health Care-Seeking Behavior (Practice)

**Figure 2:**
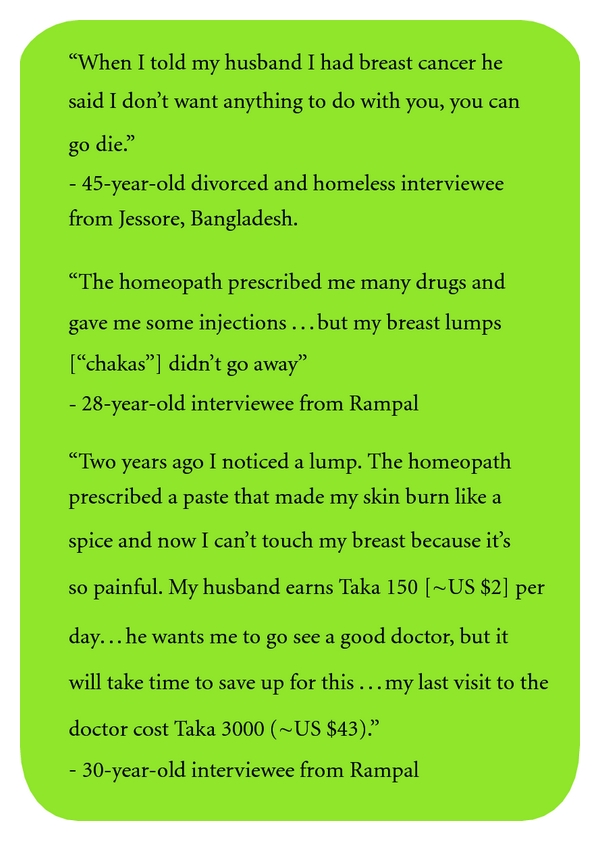


The majority of women report knowing they have a breast problem, often for a long period of time, but that they *consciously choose not to seek care*. Many reasons cited for this included mistrust of the doctor (having experienced or seen examples of bad treatment in the past), wanting to see a female doctor (women are embarrassed to show their bodies to a male doctor, yet female doctors are the exception), preferring to use alternative medicine first (homeopathic, spiritual healers, and ayurvedic), feeling too much responsibility to the family (child and elder care, cooking, and cleaning) to leave for her own care, and fears that if she is diagnosed with cancer that it will ruin her family financially or that her husband will leave her as a result. A number of women mentioned other women in their village who wanted to come for care but feared that if their husbands found out they would be abandoned. One of these women never sought care and reportedly died from her disease; another initially sought care without her husband's knowledge, but after her diagnosis she did not return for treatment and was reported to be in poor condition. Similar stories are illustrated in Figure 2.

*“It's true men are not always supportive…cancer is costly, so they won't take us to the hospital [for treatment].”*

*-Sreefaltola focus group participant.*

In summary, many women chose not to seek care for known breast problems, because they felt the options open to them really constituted “no choice.”

It should be noted, also, that many of these women are suffering from other debilitating problems such as anemia, tuberculosis, and parasites, each of which make seeking care, already arduous, much less likely due to physical weakness and poor compromised mental status.

*“An herbal “quack” doctor told me he has a patient with a breast lump he wants to bring to me, but it is impossible because the woman's husband will divorce her if he finds out. I will have to go to her home to see her.”*

*-Jessore nurse describing a conversation with an herbal doctor in her village.*

Male and female community members brought together to discuss the problem of breast cancer in Bangladesh affirmed that often a woman with cancer is viewed as “bringing a curse to the family”; separation or divorce is common in these circumstances. For families already stretched financially and physically (e.g., malnourishment), managing a complex disease such as breast cancer threatens the entire household. In these cases, the survival of the rest of the family may be at the expense of the woman who has breast cancer, particularly if a male household member also has an illness. For example, a widow breast clinic patient shared that her son sold off all the family land and left her with nothing. She now lives with her daughter. When she noticed a breast mass three years ago, she decided that she could not go for treatment because her daughter's husband was already suffering from kidney problems that they could not afford to treat.

*“The costs add up in people's minds. My wife had a caesarian section and then a thyroid nodule. Even when you are a good person these thoughts cross your mind…if the costs are too much, maybe I should separate?”*

*-Male community group discussion participant in Rampal.*

## 7. Health Care Professional Perspectives 

Discussions with doctors illuminated the numerous health system challenges they face that have a direct impact on their work. Doctors generally confirmed our data that women first present with late stage breast cancer and that family members may be the first to recognize the disease when the tumor has ulcerated and become infected, noticeable from its smell and discharge. Some physicians stated often they were “working blind” because most patients can not afford histopathological diagnosis (or further expensive testing such as tumor hormonal receptor assays), basic staging studies, and the government hospital basic costs for surgery, drugs, or private practice doctors' fees. Their inability to provide continuity of care was also noted, as patients will often “doctor shop” in the search for (what they perceive as) the “best” treatment, which is sometimes influenced by “brokers” who encourage patients to switch doctors for a commission. Doctors reported that patients who they recommended to commence chemotherapy would not return for their first cycle or would discontinue after one or two cycles due to financial constraints or other, unspecified reasons. Continuity of care is also disrupted by poor or nonexistent patient records; electronic medical record systems are not used in Bangladesh. Overcrowded hospitals, inadequate facilities for treatment (no radiotherapy or mammography equipment), difficulty scheduling operation theater time, and poor pay were also identified as challenges to providing good treatment.

Our third effort has been to try and determine what impact our attempts to create walk-in breast problem clinics and a breast treatment center has had. Here, we were prompted by a sense that we had a number of patient contacts with women who had serious breast problems, but that collectively, we were not longitudinally seeing many women with malignant disease. We put together our data about patients whom we thought had serious problems and found that of women presenting with known or suspected breast cancer cases, only a third were returning for follow-up treatment (Table 3).

Subsequently, we have been trying to tighten up these data and obtain more detailed information about specific types of treatment, reasons for not getting treatment when obvious breast cancer was present, and reasons for absence of further evaluations. Limited information confirms the issues which were identified in the interviews of patients and doctors.

Finally, as noted, we have set up primary breast problem walk in clinics and created an outpatient diagnostic and coordination of care breast center. In these facilities, we have seen more than 2000 women. Besides confirming the impressions from the foregoing assessments and data, our experiences have suggested that basic breast problem diagnosis and treatment in the division are very poor and render very limited value for the financial resources spent. Patients are urged to undergo unnecessary testing—mammography for example—or CT scanning in the absence of indications or abnormal chest X-ray or abdominal ultrasound scan finding. Excessive surgery is recommended and undertaken, histopathologic diagnosis and hormonal receptor testing are used only limitedly, hormonal therapies are significantly underutilized, and chemotherapy programs are poorly selected, suboptimally given, and rarely completed. Finally, palliative care is dismal—placebo treatments including costly chemotherapies—are overprescribed, and basic symptomatic care, such as pain relief, is rarely provided. While difficult to confirm, we often sense that economic issues on the side of health care providers play major roles in these suboptimal decisions.

## 8. Summary of Major Issues Which Govern Care for Breast Cancer in Rural Bangladesh

In pulling together our experiences and these data, it is common to talk about “barriers”, often to individuals, when collectively our perspectives have evolved to frame things as suggested in Table 4.

## 9. Discussion

Our findings support the idea that barriers to effective breast cancer management in the Khulna Division of Bangladesh are rooted in complex sociocultural, economic, and health-systems issues; most of these, including gender equity and human rights, are beyond the scope of the usual approach to cancer control but have a profound influence on effective care on a population level. Studies in other parts of South Asia and amongst immigrants to high-income countries from South Asia have suggested similarly complex findings [3, 25–31]. We believe that the general calls for “breast cancer awareness,” “early detection and mammography” and “access to drugs” are well intentioned but unlikely to result in a significant improvement on morbidity in these contexts.

The importance of addressing individual patient circumstances, community-wide beliefs and resources in developing socioculturally and resource-appropriate services cannot be overstated. However, the oft-cited barrier of “lack of awareness”, while certainly a factor in some cases, reflects an incomplete understanding of the situation for women such as those we encountered. Women, particularly young women, are all too frequently aware that they have a serious, even life-threatening problem, but face a myriad of other priorities that either prevent them from seeking care or are part of a conscious decision not to seek or continue care. The data in Table 3 strongly suggest that even having a focused facility to address breast cancer can only benefit one third of women. Individual decision-making models which regularly characterize high-income country authors' discussions of care seeking behaviors seem out of place when such strong societal forces are at work.

More broadly, we must consider the roles and value of women in society. Perhaps the “awareness” which is most lacking is the fact that healthy women are the *key* to healthy families and communities. As stated by Kristof [32], gender discrimination is lethal. In poor families, women are “triple threats”: they play central roles as breadwinners, nutrition leader/providers for all members particularly children, and as educators. But their important roles extend beyond the family: women are critical to social stability and peace: women's health is a central matter in prosperity and development.

In Bangladesh, women's problems take last priority, behind those of their male counterparts, in-laws, and other family members, whether by their own choice or theirs. The view of women as expendable, illustrated by the number of women were interviewed who were divorced or feared divorce due to disease, is destructive in a country where women make up over half the work force and have numerous household responsibilities. As summarized by Fathalla [33]: “Women are not dying because of untreatable diseases. They are dying because societies have yet to make the decision that their lives are worth saving.”

Women and doctors identified a number of structural barriers which point to inadequacies in the health system of Bangladesh. Doctor absenteeism and malpractice, reported by one study to be over 70% at smaller clinics in Bangladesh [34], and doctor malpractice (either as a result of incompetence or greed) contribute to the perception that allopathic medical care is ineffective and that cancer is “a death sentence”. Furthermore, services that should be very low cost or free have been co-opted by individuals seeking to make a profit from the circumstances surrounding an overstressed medical system, and many services required for standard breast cancer care are not affordable by the average Bangladeshi family. These findings suggest a deeper rooted problem with the health care system, problems that are outside the control of patients and requiring creative reforms to health service delivery.

The ability to address these problems efficiently and effectively requires a system that fosters collaborative thought and reduces impediments to rapidly testing and implementing viable solutions. Tedious bureaucratic systems, lack of collaboration across the health sector, and poor governance have contributed to delaying advances in health care solutions throughout South Asia [35] and must be minimized for significant progress to be made. A commitment to this aim alone could have major implications for advances and cost savings in not only breast cancer care, but many other infectious and chronic diseases as well. This governance/accountability/corruption subject (not limited to Bangladesh) is regularly, in substance, breadth, detail, and perniciousness, passed over in academic discussions of ways to improve health care outcomes.

Outside of the sociocultural and economic barriers to breast cancer care, women in most parts of South and Southeast Asia by and large have been excluded from clinical research which could shed light on the biology of disease and hosts amongst these populations and thus potentially better treatment options. With the commitment to and prioritization of quality research, including the training and resources required, research outcomes can have a direct impact on productivity, prosperity, and quality of life of people in low- and middle-income countries.

## 10. Future Needs and Conclusion

The IBCRF-Amader Gram experience in Bangladesh suggests a daunting task: engaging in the full spectrum of issues required to adequately address the problem of breast cancer (outlined in Table 4) is a multidisciplinary effort. Creative solutions undertaken in a research- and evaluation-based environment are needed if we are to obtain solutions appropriate for the diversity of populations living in low- and middle-income countries. The lessons learned from the IBCRF-Amader Gram activities, however, are the basis for a number of recommendations which may contribute to improving breast cancer outcomes globally.

1. Low- and middle-income countries need to consider models for making available low-cost treatment options, in combination with better health insurance coverage for breast cancer treatment.
2. Efforts to increase opportunities for rigorous biological and social science research and the capacity to implement such research through collaborative, multinational projects. Such projects can contribute to the development of enriched sociobehavioral models that bring cultural issues into greater consideration.
3. Low- and middle-income countries must enforce governance and more accountability of health infrastructure in order to achieve efficient and effective health outcomes.

Breast cancer control in a low-income country such as Bangladesh is a challenging endeavor influenced by a myriad of forces. Yet, with a commitment to understanding and addressing health-system needs specific to the unique conditions of a country through creative and rigorous efforts, the goal of reducing the suffering and death from breast cancer can be achieved worldwide. 

## Figures and Tables

**Table 1:** 238 consecutive new cases of breast cancer from 2007-2008 at Khulna Medical College and Hospital.

Stage I/II (local): *n* = 9 (4%); curable
Stage III+ (regionally advanced): *n* = 208 (87%); cure unlikely
Stage IV (distant metastases): *n* = 21 (9%); incurable

**Table 2:** Bangladesh hormone receptor test results pre- and posttissue processing protocol implementation.

Preprotocol implementation: 3/14 positive (21%)
Postprotocol implementation: 47/65 positive (72%)

**Table 3:** Treatment received in 245 rural Bangladeshi women with obvious or strongly suspected breast cancer.

33% (*n* = 82) received treatment of some kind
32% (*n* = 79) with obvious breast cancer had no treatment
34% (*n* = 84) with strongly suspected cancer had no further evaluations

**Table 4:** Broad themes influencing outcomes from breast cancer in rural Bangladesh.

* Health Systems*: Limited and dysfunctional facilities which dictate limited access; practitioner absenteeism; weak primary care, widely prevalent alternative care systems, fragmented care, catch as catch can versus organization structured on results and value, limited use of evidence-based care (both system and for host issues in this population of people)
*Human Rights—aka “structural violence”*: Extreme poverty; gender discrimination-cultural norm; class discrimination; market discrimination
*Societal Governance*: Lack of transparency and corruption; lack of independence of clinical medicine from pharmaceutical companies

## References

[1] Carlson RW, Anderson BO, Bensinger W (2000). NCCN practice guidelines for breast cancer. *Oncology*.

[2] The Lancet (2009). Breast cancer in developing countries. *The Lancet*.

[3] Remennick L (2006). The challenge of early breast cancer detection among immigrant and minority women in multicultural societies. *The Breast Journal*.

[4] CIA The world factbook. https://www.cia.gov/library/publications/the-world-factbook/geos/bg.html/.

[5] Bangladesh Bureau of Statistics Bangladesh population statistics. http://www.geohive.com/cntry/bangladesh.aspx/.

[6] The World Bank Country and lending groups. http://data.worldbank.org/about/country-classifications/country-and-lending-groups/.

[7] The World Bank World development indicators 2011. http://data.worldbank.org/data-catalog/world-development-indicators/wdi-2011/.

[8] Ahmed SM, Hossain MA, Chowdhury MR (2009). Informal sector providers in Bangladesh: how equipped are they to provide rational health care?. *Health Policy and Planning*.

[9] Claquin P (1981). Private health care providers in rural Bangladesh. *Social Science and Medicine. Part B*.

[10] Akter T, Islam S (2006). Dhaka medical college hospital: a diagnostic study.

[11] Zaman S (2004). Poverty and violence, frustration and inventiveness: hospital ward life in Bangladesh. *Social Science and Medicine*.

[12] The World Bank Country health system profile—Bangladesh—health resources. http://www.searo.who.int/en/Section313/Section1515_6124.htm/.

[13] Mabud MA Demographic implicatins for helath human resources for Bangladesh.

[14] Brown M, Goldie S, Draisma G, Harford J, Lipscomb J (2006). Health service interventions for cancer control in developing countries. *Disease Control Priorities in Developing Countries*.

[15] Bhurgri Y, Kayani N, Faridi N (2007). Patho-epidemiology of breast cancer in Karachi ‘1995–1997’. *Asian Pacific Journal of Cancer Prevention*.

[16] Sen U, Sankaranarayanan R, Mandal S, Ramanakumar AV, Parkin DM, Siddiqi M (2002). Cancer patterns in Eastern India: the first report of the Kolkata Cancer Registry. *International Journal of Cancer*.

[17] Wilson CM, Tobin S, Young RC (2004). The exploding worldwide cancer burden: the impact of cancer on women. *International Journal of Gynecological Cancer*.

[18] Porter P (2008). ‘Westernizing’ women’s risks? Breast cancer in lower-income countries. *The New England Journal of Medicine*.

[19] Cancer Research Fund World Breast cancer worldwide. http://www.wcrf.org/cancer_facts/women-breast-cancer.php/.

[20] Sadana R, D’Souza C, Hyder AA, Chowdhury AMR (2004). Importance of health research in South Asia. *The British Medical Journal*.

[21] World Health Organization Primary health care. http://who.int/topics/primary_health_care/en/.

[22] Harford JB (2011). Breast-cancer early detection in low-income and middle-income countries: do what you can versus one size fits all. *The Lancet Oncology*.

[23] International Breast Cancer Research Foundatio (2011). Our mission. http://www.ibcrf.org/OurMission.cfm/.

[24] Uy G (2007). Immunohistochemical assay of hormone receptors in breast cancer at the Philippine General Hospital: importance of early fixation of specimens. *Philippine Journal of Surgery and Surgical Specialties*.

[25] Ahmad F, Mahmood S, Pietkiewicz I, McDonald L, Ginsburg O Concept mapping with South Asian immigrant women: barriers to mammography and solutions.

[26] Loh S, Packer T, Yip CH, Low WY (2007). Perceived barriers to self-management in Malaysian women with breast cancer. *Asia-Pacific Journal of Public Health*.

[27] Parsa P, Kandiah M, Abdul Rahman H, Zulkefli NM (2006). Barriers for breast cancer screening among Asian women: a mini literature review. *Asian Pacific journal of cancer prevention*.

[28] Mitchell J, Lannin DR, Mathews HF, Swanson MS (2002). Religious beliefs and breast cancer screening. *Journal of Women’s Health*.

[29] Ma GX, Shive SE, Wang MQ, Tan Y (2009). Cancer screening behaviors and barriers in Asian Americans. *The American Journal of Health Behavior*.

[30] Uba L (1992). Cultural barriers to health care for Southeast Asian refugees. *Public Health Reports*.

[31] Wu T-Y, Hsieh HF, West BT (2008). Demographics and perceptions of barriers toward breast cancer screening among Asian-American women. *Women and Health*.

[32] Kristof ND (2010). *Half the Sky: Turning Oppression into Opportunity for Women Worldwide*.

[33] Fathalla MF (2006). Human rights aspects of safe motherhood. *Best Practice and Research: Clinical Obstetrics and Gynaecology*.

[34] Chaudhury N, Hammer JS (2004). Ghost doctors: absenteeism in rural Bangladeshi health facilities. *World Bank Economic Review*.

[35] Lewis M (2006). *Governance and Corruption in Public Health Care Systems*.

